# Supplementary material for: Synergistic evolution of soil microaggregates biogeochemical processes driven by elevation gradients in Tongbai Mountain
Source: PLoS One. 2025 Jun 2;20(6):e0325425. doi: 10.1371/journal.pone.0325425 (PMC12129339; doi:10.1371/journal.pone.0325425)
Supplement: Attachment — Submitted filename: Author to respond Editor - PLOS One.docx [file pone.0325425.s002.docx]

Response to Editor Comments

**Dear Editor,**

Thank you for providing us with valuable feedback and suggestions, which have significantly improved the quality of our manuscript. Here we submit a revised manuscript with the title “Synergistic Evolution of Soil Microaggregates Biogeochemical Processes Driven by Elevation Gradients in Tongbai Mountain”, which has been modified according to the suggestions carefully. The followings are point-to-point responses to the comments.

**Comment 1:** Please ensure that your manuscript meets PLOS ONE's style requirements, including those for file naming. The PLOS ONE style templates can be found at https://journals.plos.org/plosone/s/file?id=wjVg/PLOSOne_formatting_sample_main_body.pdf and https://journals.plos.org/plosone/s/file?id=ba62/PLOSOne_formatting_sample_title_authors_affiliations.pdf

**Response 1:** We have revised the manuscript according to the PLOS ONE formatting style, including the main text structure and file naming requirements. All revised files conform to the templates provided in the formatting guidelines.

**Comment 2:** In your Methods section, please provide additional information regarding the permits you obtained for the work. Please ensure you have included the full name of the authority that approved the field site access and, if no permits were required, a brief statement explaining why.

**Response 2:** We have added a statement in the Methods section indicating that field sampling was carried out with the approval of the Tongbai Mountain National Nature Reserve Administration Bureau. (line 242-243). No special permits were required as the study did not involve protected species or private land.

**Comment 3:** Thank you for stating the following financial disclosure:

National Nature Science Foundation of China [grant number 41601614].

Please state what role the funders took in the study. If the funders had no role, please state: "The funders had no role in study design, data collection and analysis, decision to publish, or preparation of the manuscript." If this statement is not correct you must amend it as needed. Please include this amended Role of Funder statement in your cover letter; we will change the online submission form on your behalf.

**Response 3:** We have included the following statement in the manuscript and cover letter: "The funders had no role in study design, data collection and analysis, decision to publish, or preparation of the manuscript."

**Comment 4a & 5:** Clarify data access restrictions or upload data to a repository. Confirm data availability.

**Response4a & 5:** There are no ethical or legal restrictions on sharing the data. All relevant raw data, including values behind graphs and statistical measures, have now been uploaded as Supporting Information files and to an open repository [repository link/DOI to be inserted upon upload]. The Data Availability Statement has been updated accordingly. All relevant data are contained within the article.

**Comment 6 & 7:** Figure 1 includes copyrighted [map/satellite] images. You must obtain permission or replace them.

**Response 6 & 7:** We have removed the previously used copyrighted image in Figure 1 and replaced it with a schematic diagram titled “Schematic diagram of vertical vegetation zonation along an altitudinal gradient in Tongbai Mountain.” The updated figure complies with the CC BY 4.0 license, and the figure caption has been revised to include appropriate attribution.

**Sincerely,**

**Chunjie LI**
