# Supplementary material for: Synergistic evolution of soil microaggregates biogeochemical processes driven by elevation gradients in Tongbai Mountain
Source: PLoS One. 2025 Jun 2;20(6):e0325425. doi: 10.1371/journal.pone.0325425 (PMC12129339; doi:10.1371/journal.pone.0325425)
Supplement: Attachment — Submitted filename: Author to respond reviewer 2 - PLOS One.docx [file pone.0325425.s003.docx]

Response to Reviewer 2 Comments

**Dear Reviewer,**

Thank you for your insightful feedback and valuable suggestions, which have greatly contributed to improving the quality of our manuscript. We are pleased to submit a revised version of our manuscript titled “Synergistic Evolution of Soil Microaggregates Biogeochemical Processes Driven by Elevation Gradients in Tongbai Mountain”. The manuscript has been carefully revised in accordance with your comments. Below are our point-by-point responses to your suggestions.

**Comment 1:** Presenting the results as 0.00±1.12 is completely unacceptable. If this content (or the content of any other element being determined) was close to the detection limit, you should write.

**Response 1:** Thank you for your valuable comment. We acknowledge that presenting results as "0.00±1.12" without specifying the detection limit (LOD) can be misleading. In our revised manuscript, we have now clearly indicated that these values are below the detection limit (LOD), and we have revised the results accordingly to reflect this. (line309-315). The relevant results are now presented as “< LOD” (below the limit of detection) to better align with scientific reporting standards for data close to the detection limit. We have made these changes in both the manuscript text and Table 3 to avoid any potential misinterpretation. We appreciate your suggestion, which helps to improve the accuracy of our presentation.

**Comment 2:** Despite the adequacy of your answer, I recommend adding information about the analysis of certified reference materials to the text. The part of your answer is quite acceptable for it.

**Response 2:** We have included details in the manuscript on the calibration of our methods using certified reference materials (GBW07401 and GBW07404 from the Chinese National Institute of Metrology), which cover major and trace element concentrations, as well as major element oxide concentrations relevant to soil analysis. The analytical accuracy was validated through repeated testing, with recovery rates typically ranging from 90% to 110%.

This information has been added to the Methodology section (lines 303-308) of the manuscript for transparency and clarity. The specific text added is as follows:

“**Calibration was carried out using certified reference materials (GBW07401 and GBW07404 from the Chinese National Institute of Metrology), which cover the major and trace element concentration ranges relevant to soil. The analytical accuracy was validated through repeated testing of these reference materials, with the results compared to their certified values, yielding recovery rates typically ranging from 90% to 110%.**”

We believe that this addition strengthens the rigor of our study and addresses your concern regarding the quality assurance of our analytical methods.

We would like to thank you for your invaluable feedback, which has helped improve the manuscript. We believe that the revisions made in response to your suggestions have significantly enhanced the quality of our work. We look forward to your further feedback.

**Sincerely,**

**Chunjie LI**
